# COVID-19 Detection from Cough Recordings Using Bag-of-Words Classifiers

**DOI:** 10.3390/s23114996

**Published:** 2023-05-23

**Authors:** Irina Pavel, Iulian B. Ciocoiu

**Affiliations:** Faculty of Electronics, Telecommunications and Information Technology, “Gheorghe Asachi” Technical University of Iasi, Bd. Carol I 11A, 700050 Iasi, Romania; irina.pavel@etti.tuiasi.ro

**Keywords:** COVID-19, cough, bag-of-words, sparse encoding

## Abstract

Reliable detection of COVID-19 from cough recordings is evaluated using bag-of-words classifiers. The effect of using four distinct feature extraction procedures and four different encoding strategies is evaluated in terms of the Area Under Curve (AUC), accuracy, sensitivity, and F1-score. Additional studies include assessing the effect of both input and output fusion approaches and a comparative analysis against 2D solutions using Convolutional Neural Networks. Extensive experiments conducted on the COUGHVID and COVID-19 Sounds datasets indicate that sparse encoding yields the best performances, showing robustness against various combinations of feature type, encoding strategy, and codebook dimension parameters.

## 1. Introduction

There are rare situations in modern times that triggered a comparable research effort as the recent COVID-19 pandemic. With more than 700 million confirmed cases [1] and about 6 million reported death toll (excess deaths estimated as high as 18 million [2]), the recent COVID-19 pandemic has severely affected all aspects of social life and still impacts our daily routine. While obtaining efficient vaccines or alternative treatment schemes for diminishing the effects of the disease has been the main target, the sudden outbreak of the pandemic generated an unprecedented effort aiming at modeling the spread of the disease, predicting the evolution of the number of cases, and offering reliable prognosis on the health status of the people.

One significant pandemic effect is reflected in the explosion of publications related to this subject. At the beginning of April 2023, the World Health Organization COVID-19 Research Database included more than 850,000 items (a small number of which were preprints) [3]. According to a study conducted on the Scopus database, more than 210,000 COVID-related papers had been published until 1 August 2021 [4]. The standard publishing patterns of the journals have been “covidised”, generating enormous pressure on the reviewers, editorial boards, and authors themselves. Fast-track mechanisms for pandemic-related papers have been established, many of the datasets have been made publicly available, and many journals experienced a massive increase in citations and associated impact factors. Nevertheless, several authors took a critical perspective on those effects, identifying the risk of “masking” the research efforts related to other health topics and raising concerns about the actual practical value (in terms of reproducibility and scalability) of the various approaches. As such, an evaluation conducted on 62 studies (selected based on quality from an initial set of 415 papers) related to COVID-19 detection from chest radiographs and CT scans concluded that “*none of the models identified are of potential clinical use due to methodological flaws and/or underlying biases*” [5]. Moreover, a recent study focusing on COVID-19 screening using audio-type data (cough, voice, breath) indicates that AI-based solutions yield no clear diagnostic improvement over decisions based on symptoms reported by the subjects under study [6].

Obtaining reliable, minimally intrusive, and affordable means of identifying COVID-19-infected people has been a significant objective hence a broad range of sensory data, equipment, and analysis methods have been thoroughly investigated. RT-PCR (PCR with reverse transcription) tests have been typically considered the principal means for confirming the infection, while inexpensive, less invasive, and more accessible alternatives have been actively searched for. Two lines of research have been mainly considered: (a) radiation-based solutions such as chest radiographs (CXT) and computer tomography (CT)—though proven effective, their frequent use is excluded for medical reasons, while additionally implying high costs and/or limited availability; (b) audio-based approaches using cough, speech, and breathing, or combinations of those. In both cases, several large, representative, diverse datasets have been compiled, many of which were made public.

Systematic reviews of existing diagnostic approaches have been published [7], while others have focused on assessing realistic performance evaluations when using specific sensory data [5,6,8]. The methodological protocols consider criteria related to possible sources of datasets bias, the use of external validation datasets, the type of calibration/validation procedures, and the diversity of diagnostic models. The reviews also include useful recommendations on data quality assessment, model evaluation, and reproducibility of the results.

Chest CT and radiograph-based solutions have been thoroughly investigated in [5], revealing that most papers use transfer learning from off-the-shelf convolutional neural architectures. At the same time, the actual number of outputs varies from simple COVID/non-COVID discrimination to multiclass setups, including viral/bacterial pneumonia, interstitial lung disease, or lung opacity. Most models use 2D imaging inputs, although some CT-oriented studies use 3D volume datasets. Several papers consider hand-crafted features (sometimes complemented by clinical information) to be processed by classical machine learning algorithms (logistic regression, random forest, Support Vector Machines). Some contributions consider preliminary lung segmentation procedures prior to the actual classification step.

The limitations, challenges, and opportunities of using audio-type data for reliable COVID-19 detection have been the subject of many publications. The sensory information includes cough, speech, breathing, or combinations of those. A number of crowd-sourced publicly available datasets have been compiled, while simple binary discrimination between healthy and infected people has been mainly targeted. An interesting paper systematically investigates the effect of the various sources of bias [9], raising concerns about the over-optimistic performances frequently reported in the literature.

The possibility of extracting relevant biomarkers from ECG recordings has also been considered [10,11], given that clinical practice revealed that COVID-19 has a detrimental effect on the cardiovascular system. Both hand-crafted and learned features, including time series-to-image conversion techniques, have been typically used, while 1D and 2D convolutional neural networks have shown the best performances.

Additional approaches include lung ultrasound (LUS) or point-of-care ultrasound (POCUS), which has advantages over CT/CXT given that it is free of ionizing radiation, low-cost, and portable [8]. A special type of vertical artifact called the B-line has been correlated with the presence of COVID-19 infection. Several publicly available datasets have been compiled, including isolated frames and video sequences. Performant-trained models include Support Vector Machines, CNNs, and hybrid CNN-LSTM architectures.

Most of the proposed solutions employ various data augmentation strategies to increase the dimensionality of the training datasets. Preprocessing steps have also proven efficient in eliminating various noise sources, artifacts, or enhancing the data quality. Generalization performances have been improved by optimal hyperparameters setting using separate validation sets, using dropout during training, and considering regularization strategies. Moreover, multi-modal approaches combining various types of input data and augmenting the sensory input with clinical information have also proven effective.

In the search for practical, reproducible, scalable solutions, a critical requirement consists in avoiding the various types of data bias that may severely affect the reliability of the reported results, yielding over-optimistic performances. Moreover, for audio-based solutions, it is worth noting that the actual method used for gathering the original data (crowd-sourced vs. within a medical facility) is of utmost importance for a realistic evaluation of the performances (reliable labeling of the recordings should be confirmed by medical specialists since self-reported information can be misleading).

Two main approaches have been considered when using audio-type input data for healthy/infected people discrimination. The first relies on hand-crafted feature extraction to be further classified using a broad range of options, including random forests, multilayer perceptrons, or support vector machines (SVM). The list of feature types reported in the literature includes low-level descriptors such as mel-frequency cepstrum coefficients or linear predictive coefficients, along with statistical functionals associated with such descriptors (e.g., means, moments, durations, extreme values) [12]. The second approach first transforms the time series into 2D representations (typically based on mel-frequency cepstrum coefficients (MFCC) spectrograms) and speculates the remarkable image classification performances of convolutional neural networks (CNNs). Custom-designed architectures may be considered, but pre-trained models have been mainly used, implementing the well-known transfer learning approach (only part of the model parameters are subject to a learning procedure, considering the specific dataset under study).

The present paper targets healthy vs. COVID-19-infected people supervised discrimination using cough recordings and builds on the previous successful use of the bag-of-words (BoW) classifier in biometric applications using ECG signals [13]. It evaluates the efficiency of various experimental setups using distinct combinations of feature extraction techniques and encoding procedures combined with a support vector machine (SVM) classifier. The performance metrics include discrimination accuracy, sensitivity, F1-score, and the area under curve (AUC). Extensive tests have been conducted using two crowd-sourced datasets, namely COUGHVID (250 subjects/class) and COVID-19 Sounds (acquired in a medical facility, 450 subjects/class). Specific preprocessing steps have been applied to clean up the recordings based on their quality and select audio segments, including only pure cough sounds. The paper extends preliminary results in [14] by including additional feature extraction strategies and encoding procedures. New developments are presented regarding the effect of both input and output fusion approaches on classification performances. Moreover, a comparative analysis against 2D solutions using Convolutional Neural Networks is also included.

## 2. Materials and Methods

Audio-based approaches to COVID-19 detection have typically considered voice, cough, and breath recordings as the primary sensory source, while some papers have also studied the effect of combining those following an input fusion approach. Many feature extraction procedures, classifiers, or end-to-end solutions have been proposed and tested on various datasets, exhibiting varied performances. Excessively optimistic ones are typically considered prone to multiple sources of bias, the effects of which have also been systematically addressed [9].

This section presents the basic principles behind the bag-of-words classifier described in [13,14] and references therein, following a description of the preprocessing methods employed for data preparation.

### 2.1. Overview of Bag-of-Words Models

Text documents analysis, more specifically, the analysis of the similarity between texts, may be performed by comparing the histograms that would count the frequency of appearance of words belonging to the same dictionary [15] without considering the order of appearance or other grammar-specific information. This intuitive principle has primarily inspired the Bag-of-words (BoW) classifier model, which has subsequently been extended to both time series and computer vision applications [16,17,18].

**Figure 1:**
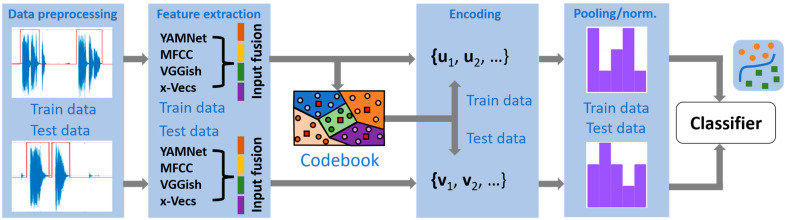
The block diagram of the COVID-19 classifier using the BoW approach.

The standard BoW processing pipeline is described in Figure 1 and includes the following modules (valid for both time series and image data):

(a) Preprocessing methods aiming at improving the quality of the data: noise/artifact removal, amplitude/sampling frequency normalization;
(b) Computation of (hand-crafted/automatically generated) feature vectors from successive temporal intervals or localized image patches from each time series/image in the original dataset. The feature vectors are obtained through a similar procedure and using the same setup parameters for the entire dataset under study. In addition, they may typically undergo a subsequent data splitting step into specific (training/test) subsets;
(c) Generating a representative codebook based on the training set feature vectors, usually employing a clustering algorithm. A powerful alternative rooted in redundant representations theory [19] may consider learning a so-called (fixed or data-dependent) dictionary, which is a matrix whose columns (termed atoms) may be used for parsimonious data representation. Online procedures for computing the codebook have also been introduced, enabling continuous updating of the codewords according to new data;
(d) Once the codebook is available, a unique or, more general, a combination of specific codewords is assigned to each training/test set feature vector, implementing an encoding procedure (both training and test sets should use the same codebook);
(e) Given the encoding of the collection of feature vectors that define each training/test time series or image, a compact representation of those is obtained through a histogram counting the frequency of codeword appearances. Since such a pooling strategy may yield histograms with largely variable dynamic ranges, a scale-normalization procedure is typically applied to enable fair comparison of the results;
(f) Finally, classification is performed based on the available histograms using specific distance measures, some of which are particularly useful when dealing with histogram-type data [20]. Nearest-neighbor, multilayer perceptrons, or Support Vector Machines (SVM) are typical classifier models considered in the literature.

### 2.2. Components of BoW Models

This section details each module of the general BoW model represented in Figure 1. We describe the preprocessing steps and the actual feature types extracted from the audio recordings. Then, we continue with the definitions and specific parameters of the various codebook generation procedures, encoding strategies, and similarity measures. The text follows the detailed presentation from [13] and references therein.

#### 2.2.1. Cough Recordings Preprocessing

The performance of the infected vs. healthy people discrimination procedure critically depends on the quality of the training data. As such, a preliminary quality check of the audio recordings is mandatory since often those are unclear, erroneously labeled as cough, or include various sources of superimposed noise. In addition, since typically, the records would consist of multiple cough episodes, the temporal localization of the actual cough segments should be first reliably identified, and the corresponding regions should be further concatenated.

Before performing any feature extraction procedure, the signals are first normalized into the [−1, 1] range and resampled to a common 16 kHz sampling rate after preliminary low-pass filtering using a Butterworth filter with an 8 kHz cut-off frequency.

**Figure 2:**
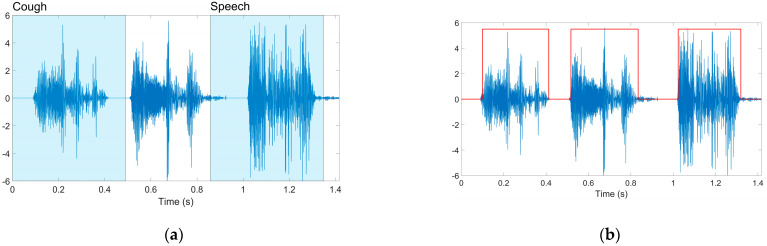
Audio record segmentation: (**a**) Cough segments erroneously classified as speech by the pre-trained YAMNet model; (**b**) Masked regions (in red) selected as cough segments by the hysteresis-based approach.

We considered two distinct options to identify the audio segments that do contain pure cough sounds. Following other studies, we first tested the efficiency of a YAMNet deep convolutional neural architecture pre-trained on the AudioSet-YouTube corpus to classify audio-type data into 521 different classes (including the “Cough” class) [21,22]. However, experiments using the pre-trained model available in MATLAB 2022a revealed inconsistent conclusions, often interpreting actual cough segments as “Throat clearing” or even “Speech”. As such, we switched to the method introduced in [23] that uses a digital hysteresis for selecting regions exhibiting rapid signal power variations specific to cough samples. The solution additionally enables the assessment of the audio quality by computing the ratio between the signal powers in the cough regions and the rest of the recordings, respectively (comparing this against a user-defined threshold value permits the selection of only reliable, consistent audio samples). As such, we selected only audio samples for which the previously defined signal-to-noise ratio exceeded 10 dB. Figure 2 presents a segmentation and labeling example.

#### 2.2.2. Feature Extraction

An extensive set of acoustic features have been considered in the literature, typically complemented by various statistical functionals computed on those. A representative example is the open-source openSMILE toolkit [12] that enables extracting several thousand features, generally further reduced using principal components analysis (PCA).

In the present paper, we have considered four distinct feature types extracted from the preprocessed segmented recordings, namely:

1. Classical mel-frequency cepstrum coefficients (MFCC) spectrograms, computed from 50% overlapping audio segments of 0.96 s. Distinct spectrograms were generated for each segment with a window size of 25 ms, a window hop of 10 ms, and a periodic Hanning window. 64 Mel bins covering the frequency range from 125 Hz to 7500 Hz were used, and after converting the mel-spectrogram into a log scale, we obtained log-mel images with 64 × 96 pixels per segment. Finally, the distinct spectrograms are concatenated along the mel bands dimension to represent the entire audio sample;
2. The following two feature types are obtained by applying the MFCC images described above as inputs to a couple of pre-trained convolutional neural network models extensively used in audio-oriented applications and reading the appropriate output of specific inner layers.
  The YAMNet model (referenced in the previous section as a means of detecting cough segments from audio recordings) may offer valuable discriminative information by intercepting the output of the last layer placed before the classification module (the layer is termed global_average_pooling2d in MATLAB 2022a). This yields a series of 1024-long feature vectors whose number of elements depends on the length of the analyzed time series.
  The second choice considers a VGGish model [18], where the EmbeddingBatch layer returns a set of 128-long feature vectors, each corresponding to 0.975 s of audio data;
3. The fourth option is represented by the so-called *x*-vectors originating from (text-independent) speaker verification applications using deep neural network embeddings [24]. Features are computed from successive 1 s audio segments and a window hop of 0.1 s, extracted from the output of the first fully-connected layer of the pre-trained model described in [24]. The resulting 512-long vectors are further reduced to a 150-long common length by linear projection using a pre-trained linear discriminant analysis matrix also available from [24].

One critical remark is worth mentioning: going back to the document analysis application that may intuitively justify the bag-of-words model, it is easy to see that when comparing two texts by counting the frequency of appearance of words from the same dictionary, the corresponding histograms will have a similar number of bins, only their amplitude will vary, according to the length (and content) of the documents. In our case, much similarly, since the various preprocessed, cough-segmented time series would typically exhibit different durations, the number of the feature vectors generated with the procedures above will vary. Since the collection of feature vectors is always encoded regarding the same set of codewords, we conclude that the encodings can accommodate variable-length recordings. The scale-normalization step described in the previous section compensates for this variability source, enabling fair comparison between recordings.

#### 2.2.3. Codebook Generation

When designing the codebook, the typical choice has been some clustering algorithm. As a classic example, *k*-means is a well-known unsupervised clustering algorithm aiming at identifying a set of prototype vectors (centroids) that compactly represent collections of data points, such as the sum of distances from the data points to the nearest cluster centers is minimized [25]. Furthermore, L1 distance can be alternatively used instead of the typical L2 choice to provide enhanced robustness to outliers. Hierarchical or multiresolution clustering approaches have also been proposed, mainly for dealing with image datasets.

Linear representations over redundant bases have gained much interest during the last decades, mainly within the sparse coding framework [26,27], and proved a viable alternative to clustering algorithms even for BoW models [13]. The method basically enables the representation of multi-dimensional vectors as a linear combination of a few columns of a dictionary matrix (those are called atoms), selected from a set of possible candidates that is much larger than the dimensionality of the vector under study. The dictionary may be chosen from a list of data-independent options well-known in the literature or following a data-dependent learning procedure to select atoms better suited to represent the signals of interest [28] parsimoniously. Similarly to [13], in the present paper we used a computationally efficient online training algorithm [29] that updates the dictionary as new data becomes available.

#### 2.2.4. Encoding Procedure

The encoding procedure is critical for the success of the BoW approach, and a broad set of solutions have been proposed. One key aspect differentiating the various options refers to the actual assignment procedure, following which a given feature vector should be coded as a single codeword (hard assignment) or a combination of those (soft assignment). In addition, as indicated in Figure 1, both training and test datasets should be subject to the same encoding procedure based on a common codebook.

Following the same notation as in [12], let us consider a collection of *M*-dimensional local feature descriptors $X\;\;=\;\;\left[x_{1},\;x_{2},\;\ldots \;x_{N}\right]\;\;\in \;{ℜ}^{M\times N}$ and a codebook of *K* codewords of the same dimensionality $D\;\;=\;\;\left[d_{1},\;d_{2},\;\ldots ,\;d_{K}\right]\;\;\in \;{ℜ}^{M\times K}$. To accommodate both single and multiple codewords encoding, we define the actual code of an input vector **x***_i_* as a *K*-dimensional vector **u***_i_*, where one or more entries are non-zero. We have considered to following options:

A. Vector Quantization (VQ) [30]:

VQ is the classic example of the hard assignment approach and sets a reference baseline against which other solutions are to be compared. VQ simply selects the nearest codeword to the given **x***_i_* vector, whereas the codebook is typically obtained by the *k*-means clustering algorithm. If considering the Euclidean distance, the encoding is given by:

(1) $$u_{ij}\;\;=\;\;\;\left\{\begin{array}{l} 1,\;\;if\;\;j\;\;=\;\;\arg\underset{j\;=\;1\ldots K\;}{\min}{\left\|x_{i}\;-\;d_{j}\right\|}^{2}\\ 0\;,\;\;\;otherwise \end{array}\right.$$

B. Soft Assignment using the *k* nearest codewords (SA-k) [31]:

Hard assignment solutions suffer from two drawbacks: (a) a given vector **x** could be very close to more than a single codeword, but the algorithm should still pick only one codeword anyway; (b) vector **x** could be very far from any component of the codebook, but a codeword should still be selected for yielding an encoding. To cope with those limitations, soft assignment solutions enable weighted combinations of (all or a limited number of) codewords. Reference [31] demonstrates the advantages of using only a subset of *k* nearest codewords to yield the corresponding encoding. The resulting SA-k algorithm is given by [31]:

(2) $$\begin{array}{l} u_{ij}\;\;=\;\;\frac{\exp\;(-\beta \;\overset{⌢}{d}(x_{i},\;d_{j}))}{\sum_{k=1}^{K}\exp\;(-\beta \;\overset{⌢}{d}(x_{i},\;d_{j}))}\;\;\;\;\;\;\;\;\\ \overset{⌢}{d}(x_{i},\;d_{j})\;\;=\;\;\left\{\begin{array}{l} d(x_{i},\;d_{j})\;\;,\;\;\;\;if\;\;\;d_{j}\;\;\in \;\;N_{k}(x_{i})\\ \;\infty \;,\;\;\;\;\;\;\;\;\;\;\;\;\;\;\;\;\;\;otherwise \end{array}\right. \end{array}$$

where $\overset{⌢}{d}(x_{i},\;d_{j})$ is a localized version of the classical Euclidean distance that considers only the *k* nearest neighbors of a data point (those define the neighborhood *N_k_*(**x***_i_*)). The smoothing hyperparameter *β* is generally obtained by cross-validation. The detailed analysis presented in [31] not only offers an interpretation of the encoding **u***_ij_* as a degree of membership of vector **x***_i_* to the (Gaussian-type cluster around) codeword **d***_j_*, but additionally elucidates the remarkable effect of the max-pooling strategy when coupled with the SA-k method.

C. Locality-constrained Linear Coding (LLC) [32]:

The LLC algorithm, similar to the sparse coding procedure described in the next paragraph, solves an optimization problem that looks for the best linear approximation of a given vector **x***_i_* by a limited number of codewords while imposing specific constraints. LLC favors the locality of the encoding, meaning that similar feature vectors should admit correlated, much similar encodings, according to [32]:

(3) $$\begin{array}{l} u_{ij}\;\;=\;\;\arg\underset{j\;=\;1\ldots K\;}{\min}{\left\|x_{i}\;-\;Du_{j}\right\|}^{2}\;\;+\;\;\lambda \;\left\|s_{i}\;⊗\;\;u_{j}\right\|\;\\ such\;\;that\;\;1^{T}\cdot \;u_{i}\;\;=\;\;1\;,\\ where\;\;s_{i}\;\;=\;\;\exp\;\left(\frac{dist\;(x_{i},\;D)}{\sigma }\right) \end{array}$$

where the symbol ⊗ denotes element-wise multiplication, and *λ* is a regularization coefficient. **s***_i_* defines a locality adaptor that weights the codewords according to their similarity to the input feature vector, while the parameter *σ* adjusts the decay speed of the adaptor and is typically optimized using cross-validation procedures.

The constraint $1^{T}\cdot \;u_{i}\;\;=\;\;1$ originates from the shift-invariant requirements of the LLC code. An additional advantage of the algorithm is that the optimization problem above admits an analytical solution that eliminates the need to use computationally intensive optimization techniques [32].

D. Sparse Coding (SC) [27]:

The method aims at identifying the sparsest linear combination of codewords from the codebook (dictionary) that exactly represent vector **x**. As such, the following optimization problem should be solved [27] (the L0-norm counts the non-zero elements of **x**):

(4) $$\underset{u}{\min}\;{\left\|u\right\|}_{0}\;\;such\;\;that\;\;x\;=\;Du$$

This problem is computationally intractable; hence more convenient convex alternatives have typically been considered by replacing the L0-norm with the L1-norm. A convex Lagrangian reformulation is the following, where the second (regularization) term reflects a priori knowledge about the solution (the *λ* parameter is set according to the noise power and the cardinality of the dictionary) [33]:

(5) $$\underset{u}{\min}\;\;\;\left\{\;{\left\|x\;-\;Du\right\|}_{2}\;\;+\;\lambda {\left\|u\right\|}_{1}\;\right\}$$

As opposed to LLC, that favors locality instead of the sparsity of the solution, SC may yield quite different encodings for similar feature vectors. Moreover, the optimization problem in Equation (5) does not admit an analytical solution, although many efficient algorithms have been proposed, including online methods that may cope with the continuous availability of new data [29].

#### 2.2.5. Similarity Measures

A Support Vector Machine (SVM) type classifier has been used in the experiments to discriminate between healthy and COVID-19-infected people. An RBF kernel of the form $K(x,x^{\prime })\;=\;e^{-\gamma {\left\|x\;-\;x^{\prime }\right\|}^{2}}$ (where *γ* is a positive scalar parameter) has been chosen to implement the well-known kernel trick that would implicitly map generally non-linearly separable data from the original space into linearly separable one in a transformed higher-dimensional space. While the distance metric exponent measuring the similarity between a pair of vectors is typically chosen as the Euclidean distance, additional classification performances may be gained when dealing with histogram-type data if particular metrics are used instead. Two typical choices are represented by the histogram intersection (HI) and chi-squared distances ($\chi^{2}$), respectively, defined as [12,20]:

(6) $$\begin{array}{l} D_{\chi_{2}}(p,\;\;q)\;\;=\;\;\sum_{k}\;\frac{{\left\|\;p[k]\;-\;q[k]\;\right\|}^{2}}{p[k]\;+\;q[k]\;+\;\varepsilon }\;\;\;\;\;\;\;\;\;\;\;(Chi-square)\\ D_{HI}(p,\;\;q)\;\;=\;\;1\;-\;\sum_{k}\max\;\left(p[k],\;\;q[k]\right)\;\;\;\;\;(Histogram\;\;intersection) \end{array}$$

## 3. Results

This section details the results of an ablation study aiming at identifying the effect of the various setup parameters and algorithmic solutions that define the BoW processing pipeline. After introducing the datasets used in the experiments, we distinctly analyze the role of the feature extraction procedure, encoding strategy, and codebook dimension. A particular target focuses on the efficiency of both input and output fusion strategies. Finally, a comparative analysis is performed against 2D approaches involving convolutional neural networks.

The discrimination procedure is based on SVM classifiers trained with the LIBSVM MATLAB toolbox [34]. The tool enables setting the optimal values of the corresponding hyperparameters based on a grid-search procedure and provides probability estimates for the classification decision.

The performance metrics include the Area Under Curve (AUC), classification accuracy, sensitivity, and F1-score. The evaluation methods include both the classical *k*-fold cross-validation procedure and external dataset validation. It is important to note that the different folds include distinct subsets of human subjects. Moreover, to realistically evaluate the classification performances on the test set, we compute 95% confidence intervals around average values using bootstrap resampling (we used 1000 samples with replacement) [35].

### 3.1. Cough Recording Datasets

Several cough recording datasets have been compiled after the pandemic outbreak that broadly differ by the number of audio files, the acquisition strategy (crowd-sourced vs. medically validated), or the nature of the sounds (cough, speech, breathing). The experiments to be reported have been conducted on two of the most frequently used datasets, namely COUGHVID (freely publicly available) [23] and COVID-19 Sounds [36] (an agreement needs to be signed prior to granting data access).

The Coughvid dataset was compiled at the Embedded Systems Laboratory (ESL) at EPFL (Switzerland) from 1 April 2020 to 1 December 2020 through a web application and considered a broad demographic and geographic variability. It includes more than 25,000 crowd-sourced recordings (more than 1100 from people self-declared as infected), 2800 of which have been labeled by four experienced physicians but not validated by the result of an RT-PCR test. It is worth noting that the project’s homepage includes code support for identifying pure cough intervals within a given audio recording using an eXtreme Gradient Boosting classifier and a collection of audio features. Moreover, to select relevant, consistent data, the authors provide support for assessing the quality of the recording: a signal-to-noise ratio is computed by comparing the signal power of concatenated cough segments and the power of the remaining background noise and further compared to a user-defined threshold value. Nevertheless, since little agreement was found between the decisions of the four specialists, the same authors released a second study using a semi-supervised learning algorithm to improve the consistency of this crowd-sourced dataset [37]. A set of 450 samples has been used in the experiments, equally balanced between the two classes. All signals were converted into a common .wav format, the original sampling frequency of 48 kHz was reduced at 16 kHz, and only recordings exhibiting a signal-to-noise ratio higher than 10 dB were selected for the experiments.

The COVID-19 Sounds dataset has been compiled by the University of Cambridge. It includes cough, breathing, and voice recordings from more than 36,000 participants covering a broad range of demographics, ages, and health conditions. The data were converted to a common .wav format with a 16 kHz sampling frequency, eliminating noisy/inconsistent recordings. A set of 900 samples has been used in the experiments, equally balanced between the two classes.

### 3.2. Effect of the Feature Extraction Procedure

**Figure 3:**
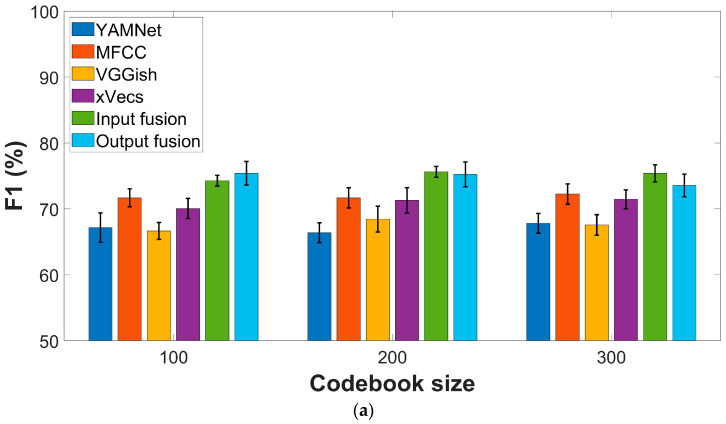

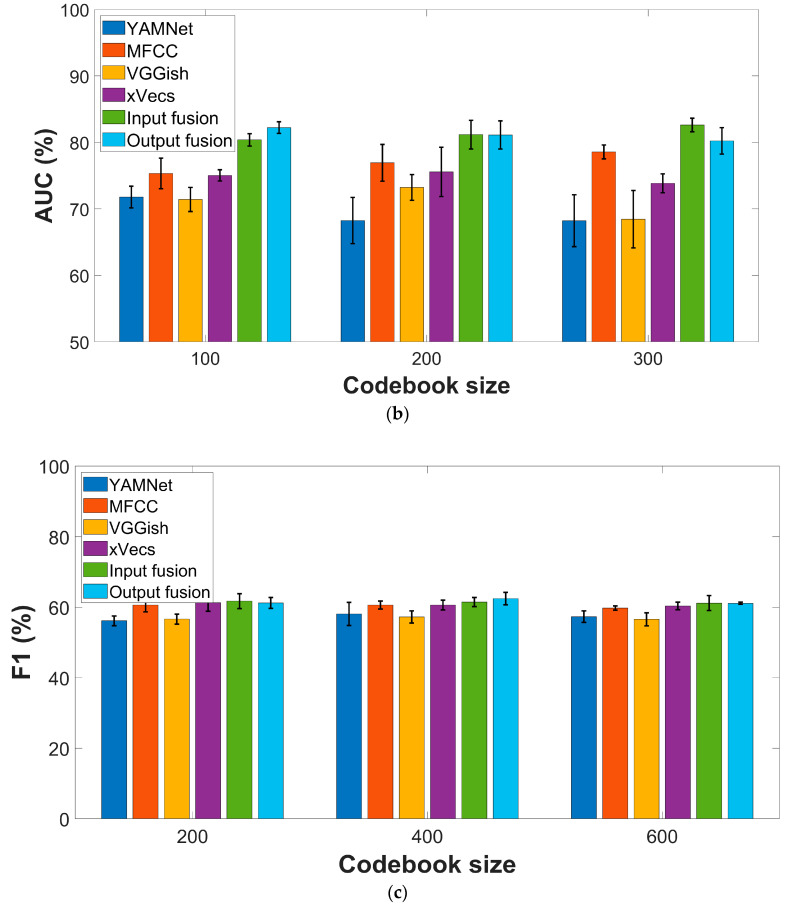

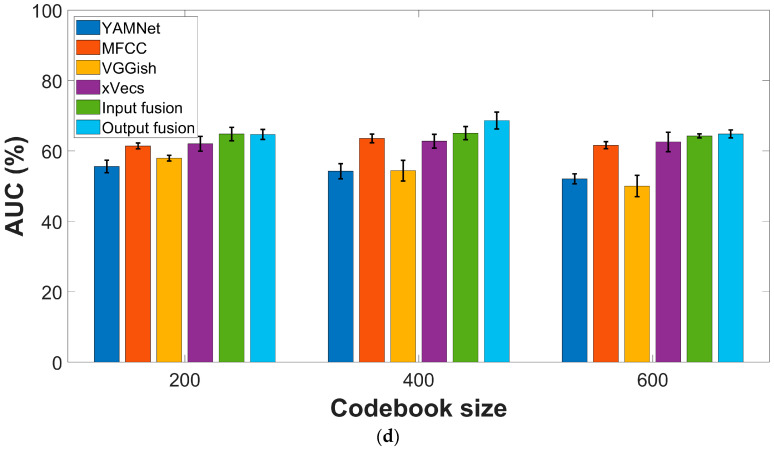
Performance metrics for various feature extraction procedures: (**a**) F1-score, COUGHVID dataset; (**b**) AUC values, COUGHVID dataset; (**c**) F1-score, COVID-19 Sounds dataset; (**d**) AUC values, COVID-19 Sounds dataset.

Figure 3 presents comparative AUC and F1 scores for the four feature extraction methods described in the previous section. All experiments considered the sparse encoding procedure based on the online algorithm introduced in [29], with a varying number of codewords. A five-fold cross-validation was performed, and each experiment was repeated ten times to compensate for possible bias in the data-splitting procedure. Performance metrics are reported regarding average values ± standard deviation across all experiments.

In the case of the COUGHVID dataset, the best F1-scores (72.25% ± 1.5) are yielded by the MFCC features, while *x*-vectors perform second best (71.45% ± 0.9). Only slight variations according to the codebook dimension are visible for all features. The AUC values show the same ordering in terms of performance, with MFCC as the top performer (78.57% ± 1), followed by *x*-vecs (75.57% ± 3.7), while no clear trend is visible according to the codebook dimension.

The conclusions are almost similar for the COVID-19 Sounds dataset, with global performances lower than those reported for the COUGHVID data. More specifically, MFCC and *x*-vecs still perform best in terms of the F1 score, with a marginal gain of the latter (61.27% ± 2.4 vs. 60.64 ± 1.3). AUC values exceed 60% only for MFCC (63.51% ± 1.2) and *x*-vecs (62.76 ± 2). The results show a relatively low variation due to the codebook dimension.

### 3.3. Effect of the Fusion Strategies

The effect of input and output fusion strategies has also been evaluated. In the former case, the distinct encodings corresponding to the four feature extraction procedures have been concatenated as a single vector to be applied as the input to the SVM classifier. In the case of the latter, we first obtain the probabilities corresponding to the classification decisions of the individual classifiers that operate on each feature type and then perform a linear weighted combination of those to provide the final discrimination decision of the output fusion approach. The weights are obtained by normalizing the individual accuracy scores according to:

(7) $$w_{i}\;\;=\;\;Acc(i)\;/\sum_{j}Acc(j)\;\;,\;\;\;i\;\;=\;\;\bar{1:4}$$

Performances reported in Figure 3 indicate the advantages of both fusion strategies. For the COUGHVID dataset, F1-scores and AUC values reach (75.62% ± 0.8) and (82.62% ± 1) respectively, for input fusion using sparse encoding. For output fusion, (75.41% ± 1.8) and (82.23% ± 0.9) values are obtained for the F1-score and AUC, respectively, using the same encoding procedure.

The improvement is also visible for the COVID-19 Sounds dataset, mainly regarding AUC performances, although not so significant as for the first dataset. For sparse encoding, F1-scores up to (61.73% ± 2.1) and (62.46% ± 1.8) are obtained for input and output fusion, respectively, while AUC fits the ranges (65.05% ± 1.9) and (68.61% ± 2.4).

### 3.4. Effect of the Encoding Procedures

**Figure 4:**
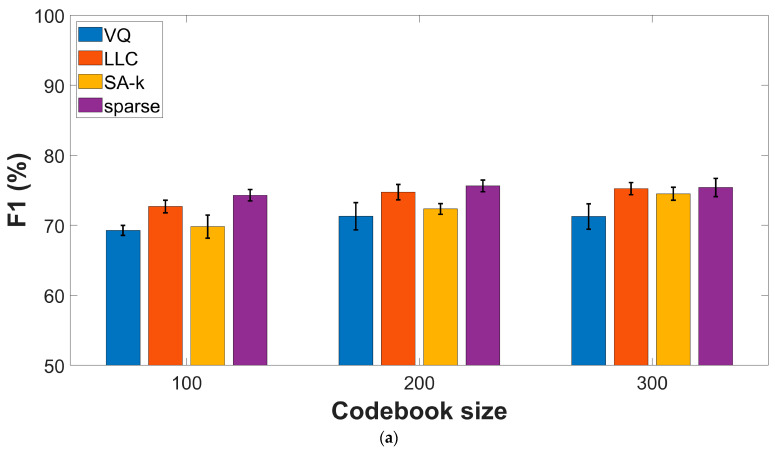

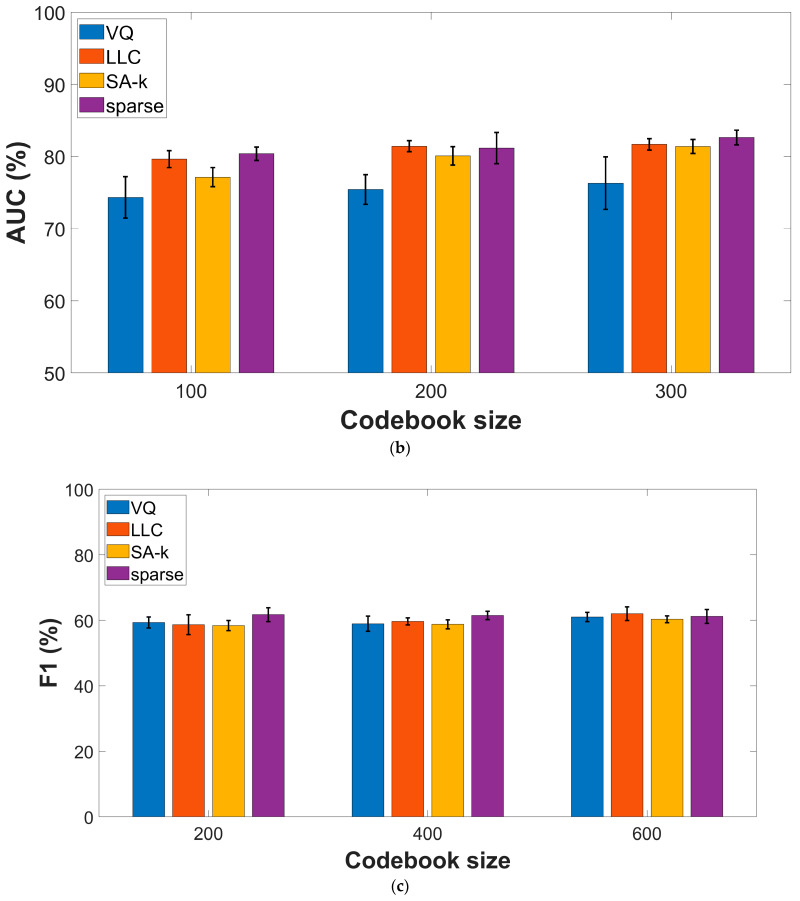

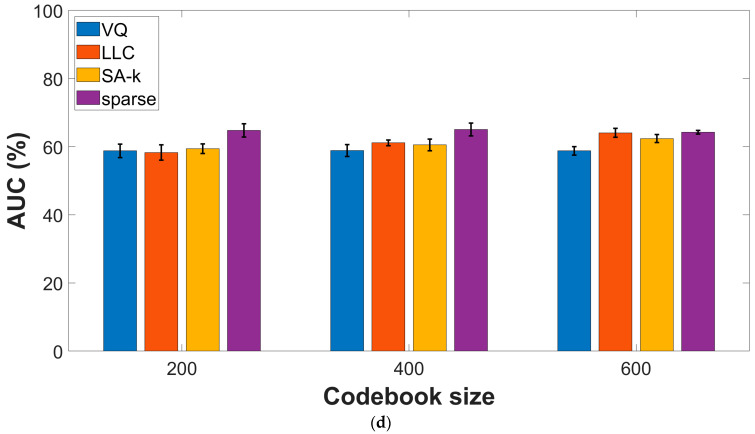
Performance metrics for various encoding procedures: (**a**) F1-score, COUGHVID dataset; (**b**) AUC values, COUGHVID dataset; (**c**) F1-score, COVID-19 Sounds dataset; (**d**) AUC values, COVID-19 Sounds dataset.

Given that the fusion approach yields superior performances when compared with the individual feature types, we decided to study the effect of the encoding procedure using data obtained by the input fusion approach. Figure 4 presents experimental results on the two datasets, indicating sparse and LLC encodings as top performers with comparable performances. For the COUGHVID dataset, F1-score and AUC values reach (75.62% ± 0.8) and (82.62% ± 1), respectively, while for COVID-19 Sounds data, we get (62.0% ± 2) and (65.05% ± 1.9), respectively.

Table 1 presents the top-3 performers for both training datasets in terms of F1-score and AUC values for specific combinations of setup parameters.

### 3.5. External Test Set Performance Evaluation

**Figure 5:**
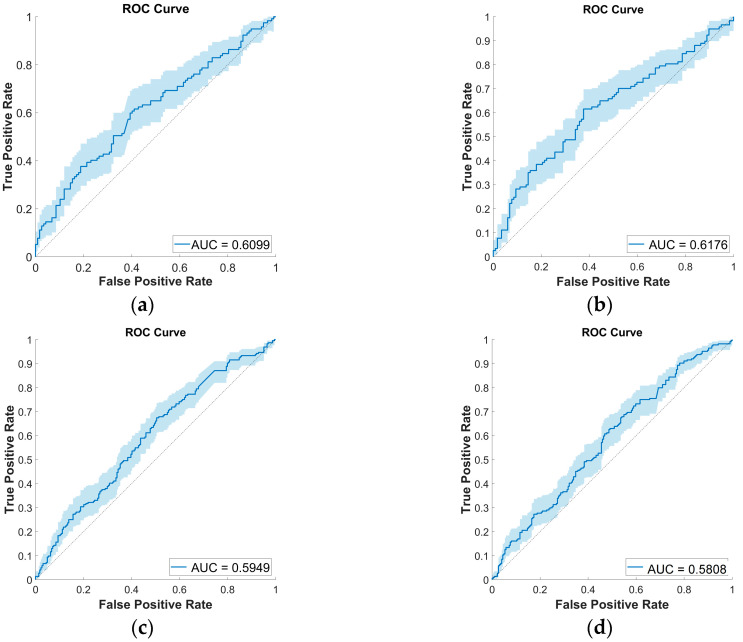
ROC curves for COVID-19 Sounds training set and external test set experiments: (**a**) Test set from COVID-19 Sounds dataset, input fusion, sparse encoding, 400 codewords; (**b**) Test set from COVID-19 Sounds dataset, output fusion, sparse encoding, 400 codewords; (**c**) Test set from COUGHVID, input fusion, LLC encoding, 400 codewords; (**d**) Test set from COUGHVID, input fusion, sparse encoding, 400 codewords.

While many papers report performance metrics using only the *k*-fold cross-validation procedure, evaluating the quality of a given classifier model using an external test set is always informative. Figure 5 presents two such scenarios in the case of the COVID-19 Sounds dataset. The first uses the same nonoverlapping train/validation/test split used in [35], where all data is extracted from the same pool of audio recordings, and demographic characteristics are balanced to avoid bias. The second experiment assesses the generalization ability of the BoW model when trained with COVID-19 Sounds data and tested on the COUGHVID dataset.

The resulting AUC values are reported for both input and output fusion strategies, using the top performers LLC and sparse encoding procedures and 400 codewords. The performances align with those obtained by the *k*-fold cross-validation method, around 61% in both scenarios, which confirms the robustness of the model.

### 3.6. Comparison with CNN Classifiers

We compare the performances of the BoW classifier with those of convolutional neural networks (CNN) models using MFCC images as inputs. As described in Section 2.2.2, classical MFCC spectrograms are computed from 50% overlapping audio segments of 0.96 s, yielding sets of log-mel images with 64 × 96 pixels per segment. However, distinct audio recordings would typically have different durations; hence we considered three different approaches to define the train and test datasets to be further applied as inputs to the various CNN models: (a) keep all MFCC images corresponding to a given recording and compute the classification decision using a majority vote: (b) randomly select a single image per audio recording; (c) the pictures generated from a given recording are averaged out to finally yield a single image per recording.

We tested three distinct CNN architectures, namely Resnet-50, MobileNet.v2, and EfficientNet-B0, that are available as pre-trained models in MATLAB 2022a. The classical transfer learning approach has been used, enabling fine-tuning of about 70% of the total number of parameters of the models. To improve the generalization capacity of the classifiers, augmentation techniques were employed by considering ±2% scale variation, ±5° rotation, and ±5 pixels horizontal and vertical translations. 5-fold cross-validation was performed, and each experiment was repeated ten times.

Results in Table 2 reveal up to 5% lower performances than the BoW model, especially for the F1-score, compared to the performances reported in Figure 3 for MFCC features. Computing the classification decision using majority voting on all images from a given recording yields the best results, while the Resnet-50 and EfficientNet-B0 architectures perform marginally better than the MobileNet.v2 option.

## 4. Discussion and Conclusions

The present paper evaluated the performances of the classical bag-of-words classifier and the role of the various design parameters on COVID-19 detection from crowd-sourced cough recordings.

It is difficult to appropriately compare the classification performances of the proposed BoW model against existing results since the experimental setup, especially the actual definition of the training/test datasets, varies to a large extent. Sources of variability include quality evaluation methods and associated threshold values for enrolling audio recordings, preprocessing and cough segmentation procedures and solutions for accommodating the variable duration of the files. Moreover, the dimensions of the training/test subsets, the possible class imbalance, or the use of augmentation techniques increase the difficulty of a relevant comparison between various proposed approaches. Finally, a significant source of concern is related to the many sources of bias that may falsely drive the results into an over-optimistic range of performance metrics. At the same time, even those are not systematically reported in a unified manner.

Comparing the results presented in Table 1 and Figure 3, Figure 4 and Figure 5 with references that consider hand-crafted features extracted from the audio recordings or similar types of features derived from CNN models [32,34,35] typically combined with SVM classifiers, we may conclude that the BoW model offers comparable or better performances in terms of F1-score and AUC values. As such, for the COUGHVID dataset, AUC values typically less than 65% are obtained. For the COVID-19 Sounds dataset, the previous references report AUC values in the 62–66% range, with only the OpenL3 + SVM model reaching 70% [38]. On the other hand, significantly better results are obtained using more sophisticated classifier models such as the Bayesian Neural Network or the Self-Supervised Audio Spectrogram Transformer [39]. Nevertheless, the same study reveals no improvement over performances based solely on user-reported symptoms, hence calling for a careful analysis of various confounders in AI-based diagnostic tools.

It is worth noting that input/output fusion techniques increase performance metrics for both datasets. For example, Figure 2 indicates up to 2.5% improvement of F1-scores and up to 5% for the AUC values compared to MFCC features, that act as second best.

While more refined architectures or fusing multiple types of audio data (cough, speech, breathing) may prove superior, the proposed approach is attractive in terms of simplicity, the ability to cope with variable duration recordings, and the possibility to adapt to newly available data if online encoding algorithms are used.

The present paper extends preliminary results [14] by considering an extended range of setup parameters for the BoW classification model. The positive effect of the input/output fusion strategies is also revealed, while the comparison with CNN-based approaches complements the analysis framework.

The experimental results show that the BoW approach is competitive against existing solutions exhibiting comparable complexity, and further work may reveal improved performances. We intend to consider appropriate augmentation techniques, enlarged datasets, and a broader range of feature extraction procedures. An interesting idea worth testing concerns the inclusion of the BoW classifier into an end-to-end learning model that would automatically compute relevant, discriminative features.

Further study of image-based classifiers, including optimized architectures using AutoML techniques, is also necessary. Moreover, the bag-of-visual words model is also worth considering for further work.

## Figures and Tables

**Table 1:** Best classification performances of BoW models.

Dataset	Model	Codewords	Accuracy (%)	Sensitivity (%)	F1 (%)	AUC (%)
COUGHVID	input fusion, sparse encoding	300	72.7 ± 1.3	61.8 ± 1.1	75.4 ± 1.3	82.6 ± 1
	output fusion, sparse encoding	100	74.3 ± 2	70.2 ± 4.3	75.4 ± 1.8	82.2 ± 0.9
	output fusion, LLC encoding	300	74.1 ± 1.9	71.4 ± 2	74.8 ± 1.8	81.4 ± 1.6
COVID-19 Sounds	output fusion, sparse encoding	400	63.2 ± 1.5	65.3 ± 1.1	62.4 ± 1.7	68.6 ± 2.4
	input fusion, sparse encoding	400	60.3 ± 1.8	56.8 ± 3.7	61.4 ± 1.3	65 ± 1.9
	input fusion, LLC encoding	600	58.7 ± 1.7	49.4 ± 2.2	62 ± 2	64 ± 1.3

**Table 2:** Classification performances of CNN models.

Dataset	Model	Accuracy (%)	Sensitivity (%)	F1 (%)	AUC (%)
COUGHVID	All frames				
	Resnet-50	68.81	66.27	69.29	74.72
	MobileNet.v2	67.12	63.05	68.36	71.88
	EfficientNet-B0	69.06	68.13	68.35	76.08
	Random frame				
	Resnet-50	68.91	58.09	64.92	76.89
	MobileNet.v2	66.09	63.09	64.10	73.06
	EfficientNet-B0	67.36	61.91	65.04	73.65
	Average frame				
	Resnet-50	66.95	65.45	66.23	72.96
	MobileNet.v2	65.41	60.55	66.88	71.82
	EfficientNet-B0	68.27	67.09	67.76	75.25
COVID-19 Sounds	All frames				
	Resnet-50	53.53	53.43	53.38	55.53
	MobileNet.v2	53.50	48.13	50.83	55.65
	EfficientNet-B0	52.47	50.06	51.31	54.01
	Random frame				
	Resnet-50	55.71	52.89	53.86	58.64
	MobileNet.v2	54.33	55.11	54.43	56.93
	EfficientNet-B0	55.56	51.19	53.28	58.17
	Average frame				
	Resnet-50	57.93	51.55	54.87	62.39
	MobileNet.v2	54.14	48.95	50.81	56.04
	EfficientNet-B0	56.07	44.41	50.07	58.43

## Data Availability

Not applicable.
